# Causes and Outcome of Systemic Inflammatory Response Syndrome in Dogs: An Observational Study of 172 Cases

**DOI:** 10.1111/vec.70143

**Published:** 2026-08-12

**Authors:** Myriam A. Knopf, Christiane Weingart, Roswitha Merle, Teresa Sandbrink, Antina Lübke‐Becker, Marcus Fulde, Caroline Robé, Marianne Skrodzki, Uwe Rösler, Stefan Schwarz, Barbara Kohn

**Affiliations:** ^1^ Small Animal Clinic Freie Universität Berlin Berlin Germany; ^2^ Institute of Veterinary Epidemiology and Biostatistics Freie Universität Berlin Berlin Germany; ^3^ Veterinary Center for Resistance Research (TZR) Freie Universität Berlin Berlin Germany; ^4^ Institute of Microbiology and Epizootics Freie Universität Berlin Berlin Germany; ^5^ Institute of Animal Hygiene and Environmental Health Freie Universität Berlin Berlin Germany

**Keywords:** canine, critical care, multiple organ dysfunction syndrome, organ dysfunction, sepsis

## Abstract

**Objective:**

To evaluate the clinical characteristics, underlying diseases, organ dysfunctions, and survival of dogs with systemic inflammatory response syndrome (SIRS) or sepsis.

**Design:**

Prospective observational study from January to December, 2023.

**Setting:**

Single‐center, veterinary university teaching hospital.

**Animals:**

One hundred sixty‐six dogs; six dogs with distinct cases of SIRS were included twice (172 cases).

**Interventions:**

None.

**Measurements and Main Results:**

Dogs were included if they met two or more of the four SIRS criteria (hypothermia or fever; tachycardia; tachypnea; leukocytosis, leukopenia, or left shift) and had a disease causing systemic inflammation. Cases were categorized into groups (G) “nonseptic SIRS” (G1: *n* = 73), “sepsis” (G2: *n* = 92), “sepsis without organ dysfunction” (G2a: *n* = 25), “severe sepsis” (G2b: *n* = 59; dysfunction of one or more organ systems), “septic shock” (G2c: *n* = 8; hypotension despite adequate fluid resuscitation), and “SIRS of unclear origin” (G3: *n* = 7). The most common causes of SIRS/sepsis were gastrointestinal diseases (*n* = 61 [35.5%]), vector‐borne diseases (*n* = 28 [16.3%]), and neoplasia (*n* = 22 [12.8%]). There was dysfunction of one or more organs in 114 cases (66.3%). The coagulation system was most frequently affected (*n* = 78 [45.3%]), followed by the cardiovascular system (*n* = 47 [27.3%]), respiratory tract (*n* = 14 [8.1%]), kidney (*n* = 13 [7.6%]), and liver (*n* = 6 [3.5%]). Overall, 128 of 165 cases (77.6%) survived until discharge (short‐term), and 116 of 159 (73%) survived ≥4 weeks after discharge (long‐term). Differences in outcome were found between G1 and G2 (short‐term: *p* = 0.046, long‐term: *p* = 0.015), with dogs in G2 having better survival. Short‐term mortality was higher in cases with one or more organ dysfunctions than in cases without (*p* = 0.031).

**Conclusions:**

SIRS can be triggered by a variety of diseases in dogs. Strict and regular monitoring is important for early detection of organ dysfunction.

**Abbreviations**
cCRP: canine C‐reactive protein
CI: confidence interval
nSIRS: nonseptic SIRS
OR: odds ratio
PCR: polymerase chain reaction
RI: reference interval
SIRS: systemic inflammatory response syndrome

## 1 Introduction

Systemic inflammatory response syndrome (SIRS) is described as a dysregulated, life‐threatening host response to a stressor [1, 2, 3, 4]. The initial criteria for diagnosing SIRS were established in human medicine in 1992 and include clinical and laboratory parameters [5]. These criteria were subsequently adapted and modified for application in dogs and cats [6, 7]. The causes of SIRS are heterogeneous and can be of infectious (e.g., bacterial, viral, fungal, or protozoal) or noninfectious (e.g., pancreatitis, neoplasia, or heat stroke) origin [1, 3, 5]. At the 1992 consensus conference in human medicine, infection‐induced SIRS was defined as sepsis [5].

A variety of pathophysiologic mechanisms common in systemic inflammation (e.g., damage of the glycocalyx and increased permeability of the blood vessels, reduced vascular responsiveness to vasopressors, impaired cellular ability to use oxygen) [8, 9, 10, 11, 12] can lead to organ dysfunction in critically ill patients. Sepsis in people complicated by organ dysfunction, hypoperfusion, or sepsis‐induced hypotension was defined in 1992 as severe sepsis, and septic shock was defined as sepsis with hypotension persisting despite adequate fluid resuscitation [5]. Since their establishment, these definitions have also been applied in veterinary medicine [1]. New definitions were established in human medicine in 2016 [4], at which time sepsis was defined as life‐threatening organ dysfunction caused by a dysregulated host response to an infection [4]; the term *severe sepsis*, thus, became superfluous. Before now, no consensus on using these new criteria in veterinary medicine had been reached [13].

SIRS and sepsis are common conditions in human medicine and are accompanied by high mortality and healthcare costs [14, 15, 16]. In 2017, almost 49 million human adults worldwide were estimated to have developed sepsis, and 11 million sepsis‐associated deaths were documented [15]. Less information is available about the incidence of SIRS and sepsis in veterinary medicine [1, 13]. In a recent study, 79.8% of the dogs presenting to an emergency room fulfilled the clinical and laboratory parameters commonly used for diagnosing SIRS [17]. However, no clinical review was performed in this population to determine whether SIRS was actually present [17]. Mortality rates in recent veterinary studies ranged from 31% to 60% for nonseptic SIRS (nSIRS) [18, 19, 20, 21] and from 12.5% to 52% for sepsis [18, 19, 20, 21, 22, 23, 24]. Organ dysfunction is a common problem in people and animals with SIRS or sepsis that leads to greater mortality [22, 23, 24, 25]. Multiple organ dysfunction syndrome can be diagnosed in critically ill people and animals if at least two organ systems are altered in function and interventions are necessary to maintain homeostasis [5, 24].

In people and dogs, early recognition and initiation of treatment are essential to improve patient outcomes [26, 27, 28, 29]. The aim of the current study was to describe the clinical characteristics of a group of dogs with SIRS of heterogeneous origins. Underlying causes of SIRS and sepsis were investigated and compared with regard to patient outcome. Furthermore, organ dysfunctions were evaluated, and possible factors influencing the outcome were studied.

## 2 Materials and Methods

### 2.1 Study Design and Study Population

This prospective observational study was conducted at the Small Animal Clinic of the Freie Universität Berlin, Berlin, Germany. No written informed consent was obtained from the animals’ owners because patient treatment was not modified for the study, and no patients or clients were identifiable. All dogs presenting to the clinic between January 2023 and December 2023 that were diagnosed with SIRS were included in the study. The cases were identified by analysis of all patients presenting to the clinic during regular service hours and through internal discussions of new cases. Data to be recorded were collected by the first author or by review of the medical record and feedback with the responsible clinician. Dogs with SIRS of different causes could be included multiple times and were evaluated as separate cases, except for the evaluation of sex and breed. Signalment of the study population was compared with that of the overall canine patient population of the clinic in 2023 to reveal possibly predispositions for SIRS. The day of SIRS diagnosis was considered Day 0.

### 2.2 SIRS Criteria and Groups

SIRS was diagnosed if two or more SIRS criteria were fulfilled in the presence of an underlying disease that triggers systemic inflammation (Table 1). Cases for which not all SIRS criteria could be assessed (e.g., due to missing hematology or other values) were excluded from the evaluation and statistical analyses concerning the number of fulfilled SIRS criteria. For the evaluation and statistical analyses regarding the frequency and severity of the individual SIRS criteria, all cases with available values were included. The day of SIRS diagnosis was defined as Day 0. Based on the 1992 human definitions [5], cases were split into the following groups (G): “nonseptic SIRS” (G1), “sepsis” (G2) (“sepsis without organ dysfunction” [G2a], “severe sepsis” [G2b], “septic shock” [G2c]), and “SIRS of unclear origin” (G3).

**TABLE 1:** Criteria for diagnosing SIRS in dogs, modified according to Okano et al. [6] and Hauptman et al. [7].

SIRS in dogs was defined as the presence of two or more of the following criteria along with an appropriate underlying disease	
1. Hypothermia or fever (°C)	≤37.9 or ≥39.0
2. Tachycardia (heart rate [/min])	≥156
3. Tachypnea (respiratory rate [/min])	≥36
4. Leukopenia or leukocytosis	(WBCs [× 10^9^/L{cells/µL}]) ≤6 (≤6000) or ≥12 (≥12,000) or left shift (immature forms [× 10^9^/L{cells/µL}]) ≥ 0.5 (500)

Abbreviation: SIRS, systemic inflammatory response syndrome.

Sepsis was defined as SIRS caused by a confirmed or highly suspected infection [5, 30, 31]. Therefore, cases were classified as septic if an infectious agent was definitively identified, or an infection was assumed due to the history, clinical examination, and the pathogenesis of the disease. This applied to patients with diseases that led to damage to the intestinal mucosa, so that bacterial translocation must be assumed [32, 33, 34], or diseases that are usually primarily or secondarily associated with a bacterial infection, such as pyometra or cholangitis/cholecystitis [35, 36, 37].

### 2.3 Diagnosis of Underlying Diseases

Information from the medical history, medical records collected from the referring veterinarian (if available), clinical examination results, laboratory findings (e.g., blood examinations, microbiological results, cytology, serology), and other diagnostic test results (e.g., imaging) were acquired for each case, and, if possible, the SIRS‐triggering disease was identified. The underlying diseases were divided into the following categories: abscess/wound, anaphylaxis, (auto)immune disorder, catheter‐associated infection, gastrointestinal tract, (known or highly suspected) neoplasia, respiratory tract, urogenital tract, vector‐borne diseases, and others. Some of the dogs were diagnosed with more than one disease. For the classification into the disease groups, the disease believed to be the trigger of SIRS was chosen. Classification was made at the time of diagnosis and, in some ambiguous cases, after resolution of the acute illness period by discussion with the senior clinician.

### 2.4 Organ Dysfunction

Organ dysfunctions were recorded if they developed as part of the disease and were either present on Day 0 or developed during hospitalization. Five organ systems were analyzed regarding dysfunction: the cardiovascular system, the coagulation system, the kidney, the liver, and the respiratory tract (Table 2). For the statistical analysis of the effects of the number of impaired organs, only the cases with a functional impairment of at least one organ were included. The final assessment was made after completion of the inpatient period.

**TABLE 2:** Definitions for organ dysfunction used for 172 cases of canine SIRS or sepsis, modified according to Lakhdir et al. [63], Kenney et al. [24], Sigrist et al. [64], and the International Renal Interest Society guideline recommendations for grading of acute kidney injury in dogs and cats.

Organ(system)	Definition
Cardiovascular system	a) Compensated shock (tachycardia, reddened mucous membranes, shortened capillary refill time, and/or bounding pulse) b) Decompensated shock (tachycardia or bradycardia, pale mucous membranes, prolonged capillary refill time, cold limbs or hypothermia, and/or hypotension or weak pulse) c) Hemodynamically relevant arrhythmia or echocardiographic evidence of endo‐/myocarditis
Coagulation system	Thrombocytopenia (<100 × 10^9^ platelets/L [<100 × 10^3^/µL]) and/or prolonged activated partial thromboplastin time and/or prothrombin time more than 25% above the upper reference limit
Kidney	Acute azotemia or increase in serum creatinine under infusion therapy by ≥26.4 µmol/L (≥0.3 mg/dL) in 48 h
Liver	Altered liver function parameters and increased liver enzyme activities
Respiratory tract	Tachypnea/dyspnea accompanied by changes of the lung on radiographs or need for supplementation of oxygen

Abbreviation: SIRS, Systemic Inflammatory Response Syndrome

### 2.5 Blood Pressure Measurement

In most cases, blood pressure was measured with Doppler^1^; however, blood pressure was measured oscillometrically^2^ in a few cases. Hypotension was defined as systolic blood pressure ≤90 mm Hg.

### 2.6 Hematology, Clinical Chemistry, and Coagulation

On Day 0, hematology was performed in 168 of the 172 cases^3^^,^ 4 ^,^ 5 ^,^ 6. A manual WBC differential blood count was performed in 95 cases, with smears stained^7^^,^ 8 and analyzed microscopically. Clinical biochemistry panel was performed in 163 cases with varying measured parameters due to indication and emergency service times^9^^,^ 10 ^,^ 11 ^,^ 12 ^,^ 13. Activated partial thromboplastin time^14^ and prothrombin time^15^ were determined in 66 cases using a coagulometer. Blood test results from the referring veterinarian were also included. Further parameters (e.g., bile acids, cobalamin, cortisol) were analyzed by an external reference laboratory.

### 2.7 Imaging and Further Examinations

Thoracic and abdominal radiographs^16^, abdominal sonography^17^, echocardiography^18^, and electrocardiograms^19^^,^ 20 were performed if indicated. Hemodynamic relevance of arrhythmias was determined on a case‐by‐case basis, taking into account type and severity. Cytologic slides (e.g., fluids or fine‐needle aspirate samples) were stained^21^ and analyzed microscopically. Urinalyses were performed using test strips^22^ and a refractometer^23^. Urine was centrifuged, and the sediment was analyzed microscopically.

### 2.8 Microbiological Examinations and Serology

Microbiological testing (e.g., of blood, urine, abdominal fluid, feces, wounds, vaginal discharge) was carried out at the Institute of Microbiology and Epizootics of the Freie Universität Berlin, Berlin, Germany^24^^,^ 25 ^,^ 26 ^,^ 27. Polymerase chain reaction (PCR) analyses and serological testing for various bacteria, viruses, and parasites were performed at external reference laboratories. Further PCR or serological examinations provided by the referring veterinarians from different laboratories were also evaluated.

### 2.9 Treatment

Both inpatient and day‐patient care were defined as hospitalization. Cases that were ultimately referred to another hospital for treatment were excluded from statistical analyses regarding the length of hospitalization. All drugs with analgesic effects were evaluated as analgesics in the following, regardless of (additional) use as antipyretics (e.g., metamizole) or sedatives (e.g., butorphanol). Antimicrobial pretreatment was defined as antimicrobial administration within the 7 days [23, 38, 39] prior to SIRS diagnosis (or in the past 14 days if treated with cefovecin [40]). Due to different underlying diseases, treatment was not standardized for all patients and was instead determined based on in‐house treatment protocols and guidelines at the discretion of the responsible clinician.

### 2.10 Outcome

Outcome was divided into short‐term (until discharge) and long‐term (until ≥4 weeks after discharge) survival. Long‐term survival was assessed by follow‐up appointments or telephone calls with the patients’ owners. Euthanized dogs were classified as euthanasia due to poor prognosis or euthanasia due to financial restrictions. Dogs that were euthanized due to financial restrictions or were discharged against medical advice were excluded from outcome analyses. Dogs for which follow‐up was not possible were excluded from analyses regarding long‐term survival.

### 2.11 Statistical Analyses

Statistical analyses were performed using a computer program for statistical calculations^28^. Data were assessed for normal distribution using histograms, and nonnormally distributed data are described as median (range). Signalment data (e.g., age, sex, breed) of the study population were compared with the data of the overall clinic canine patient population in the same year using medians and percentages. Pearson *χ* ^2^ or Fisher exact tests (when 25% or more of the cells had expected values below 5) were used to examine the association between two nominal variables. The influence of certain metric variables on the probability of organ dysfunction and survival was tested using logistic regression models. ANOVA, including Bonferroni correction for pairwise comparisons, was used to investigate the influence of the groups/subgroups on the number of SIRS criteria fulfilled and selected laboratory values. A *p*‐value of ≤0.05 was assumed to be statistically significant. The graphical representation was created using another computer program^29^.

## 3 Results

### 3.1 Signalment

One hundred seventy‐two cases of SIRS in 166 dogs were included; six dogs were included twice for distinct cases of SIRS on different hospital visits. The study population included 87 male dogs (52.4%), 29 (33.3%) neutered and 58 (66.7%) intact, and 79 female dogs (47.6%), 35 (44.3%) neutered and 44 (55.7%) intact. The median age was 7.8 years (range: 2 months to 16.9 years); however, the age of three dogs was unknown. The median weight was 16.3 kg (range: 0.9–53.0 kg), with the weight unknown in two dogs. Most dogs were purebred (*n* = 115 [69.3%]); the most frequently represented breeds were Labrador Retriever (*n* = 8), Chihuahua (*n* = 7), Golden Retriever (*n* = 6), Pomeranian (*n* = 6), Poodle (*n* = 6), German Shepherd Dog (*n* = 5), Australian Shepherd (*n* = 4), Bernese Mountain Dog (*n* = 4), Bolonka Zwetna (*n* = 4), and Rhodesian Ridgeback (*n* = 4). A total of 51 dogs (30.7%) were mixed breeds. The distribution of sex, neuter status, median age, and distribution of breed and mixed‐breed dogs of the study population was similar to the overall canine patient population of the clinic during the same year.

### 3.2 SIRS Criteria and Group Classification

All four SIRS criteria were evaluable in 151 cases (87.8%). In most cases (*n* = 73 [48.3%]), two SIRS criteria were fulfilled. Three criteria were fulfilled in 60 cases (39.7%), and all four criteria were fulfilled in 18 cases (11.9%). Leukocytosis, leukopenia, or left shift was the most commonly fulfilled criterion (*n* = 160/170 [94.1%]), followed by abnormalities in temperature (*n* = 148/172 [86%]), respiratory rate (*n* = 91/159 [57.2%]), and heart rate (*n* = 44/171 [25.7%]).

The “nSIRS” group (G1) consisted of 73 cases (42.4%). Ninety‐two cases (53.5%) were diagnosed with sepsis (G2), 59 (64.1%) of which were diagnosed with severe sepsis (G2b) and 8 (8.7%) with septic shock (G2c). Seven cases (4.1%) were categorized as “SIRS of unclear origin” (G3) (Table 3). There was no difference in the number of SIRS criteria fulfilled between G1 and G2 (ANOVA, *p* = 0.931) or G2a, G2b, and G2c (ANOVA, *p* = 0.085). A difference was found in the presence of leukocytosis (Pearson *χ* ^2^ test, *p* < 0.001) and leukopenia (Pearson *χ* ^2^ test, *p* < 0.001) between cases assigned to G1 and G2. Leukocytosis was more often found in G1 than in G2 (odds ratio [OR]: 4.62, 95% confidence interval [CI]: 2.3–9.4) and leukopenia more often in G2 than G1 (OR: 7.28, 95% CI: 3.0–17.6). Furthermore, statistically significant differences in the occurrence of leukocytosis (Fisher exact test, *p* = 0.018; G2a vs. G2b OR: 3.54, 95% CI: 1.28–9.84, G2a vs. G2c OR: 7.30, 95% CI: 1.17–45.45, G2b vs. G2c OR: 2.96, 95% CI: 0.38–11.11) and leukopenia (Fisher exact test, *p* = 0.004; G2a vs. G2b OR: 5.52, 95% CI: 1.69–18.18, G2a vs. G2c OR: 8.33, 95% CI: 1.39–49.87, G2b vs. G2c OR: 1.51, 95% CI: 0.33–6.88) were detected between cases assigned to G2a, G2b, and G2c. Leukocytosis was most likely found in G2a and leukopenia in G2c.

**TABLE 3:** Presentation of the underlying diseases (sorted into categories) of 172 cases of SIRS or sepsis in dogs split into groups based on underlying condition and classification of SIRS or sepsis.

		Abscess/wound	Anaphylaxis	(Auto)‐immune disorders	Catheter‐associated infection	Gastrointestinal tract	Neoplasia	Respiratory tract	Urogenital tract	Vector‐borne diseases	Others
		*n* = 9	*n* = 7	*n* = 14	*n* = 2	*n* = 61	*n* = 22	*n* = 7	*n* = 10	*n* = 28	*n* = 12
G1	*n* = 73	2	6	12	0	24	21	2	2	0	4
	%	2.7	8.2	16.4	0	32.9	28.8	2.7	2.7	0	5.5
G2	*n* = 92	7	1	2	2	35	1	5	8	28	3
	%	7.6	1.1	2.2	2.2	38.0	1.1	5.4	8.7	30.4	3.3
G2a	*n* = 25	6	1	2	2	9	0	1	2	1	1
	%	24.0	4.0	8.0	8.0	36.0	0	4.0	8.0	4.0	4.0
G2b	*n* = 59	1	0	0	0	20	1	4	5	26	2
	%	1.7	0	0	0	33.9	1.7	6.8	8.5	44.1	3.4
G2c	*n* = 8	0	0	0	0	6	0	0	1	1	0
	%	0	0	0	0	75.0	0	0	12.5	12.5	0
G3	*n* = 7	0	0	0	0	2	0	0	0	0	5
	%	0	0	0	0	28.6	0	0	0	0	71.4

*Note*: G1 = nonseptic SIRS; G2 = sepsis; G2a = sepsis without organ dysfunction; G2b = severe sepsis (sepsis with organ dysfunction); G2c = septic shock; G3 = SIRS of unknown origin.

Abbreviation: SIRS, systemic inflammatory response syndrome.

### 3.3 Underlying Diseases

Gastrointestinal diseases were the most common causes of nSIRS (*n* = 24 [32.9%]) and sepsis (*n* = 35 [38.0%]). Confirmed or highly suspected neoplasia was the second most frequent cause for nSIRS (*n* = 21 [28.8%]), followed by (auto)immune disorders (*n* = 12 [16.4%]). Vector‐borne diseases were the second most frequent cause for sepsis (*n* = 28 [30.4%]), followed by diseases of the urogenital tract (*n* = 8 [8.7%]) (Table 3).

### 3.4 Organ Dysfunction

There was dysfunction of one or more organ systems in 114 (66.3%) cases. One organ system was affected in 76 of 172 cases (44.2%). Multiple organ dysfunction syndrome was present in 38 cases (22.1%), with 2 affected organ systems in 34 cases (29.8%), 3 affected organ systems in 2 cases (1.2%), and 4 organ systems in 2 cases (1.2%). The coagulation system (*n* = 78 [45.3%]) was most frequently affected, followed by the cardiovascular system (*n* = 47 [27.3%]), the respiratory tract (*n* = 14 [8.1%]), the kidney (*n* = 13 [7.6%]), and the liver (*n* = 6 [3.5%]) (Table 4). Logistic regression found that meeting more SIRS criteria corresponded with a higher probability of organ dysfunction (*p* = 0.017). The probability of organ dysfunction was 1.96 times higher if one additional SIRS criterion was met (95% CI: 1.13–3.42). No difference was found in the occurrence of organ dysfunction between G1 and G2 (Pearson *χ* ^2^ test, *p* = 0.069; OR: 1.87, 95% CI: 1.0–3.6). Organ dysfunction was present in 43 of 73 cases with nSIRS (58.9%), in 67 of 92 cases with sepsis (72.8%), and in 4 of 7 cases with SIRS of unclear origin (57.1%).

**TABLE 4:** Association among the presence of organ dysfunction, the number and type of organ system(s) affected and outcome of 172 cases of SIRS or sepsis in dogs.

			Short‐term survival				Long‐term survival			
			Yes *n*	No *n*	Excluded *n*	*p*‐value	Yes *n*	No *n*	Excluded *n*	*p*‐value
Organ dysfunction	Yes	*n* = 114 (%)	80 (72.7)	30 (27.3)	4	0.047	73 (68.2)	34 (31.8)	7	0.059
	No	*n* = 58 (%)	48 (87.3)	7 (12.3)	3		43 (82.7)	9 (17.3)	6	
Number of organ dysfunctions	1	*n* = 76 (%)	59 (80.8)	14 (19.2)	3	0.019	53 (75.7)	17 (24.3)	6	0.022
	2	*n* = 34 (%)	19 (57.6)	14 (42.4)	1		19 (57.6)	14 (42.4)	1	
	3	*n* = 2 (%)	1 (50)	1 (50)	0		0	2 (100)	0	
	4	*n* = 2 (%)	1 (50)	1 (50)	0		1 (50)	1 (50)	0	
Cardiovascular	Yes	*n* = 47 (%)	28 (62.2)	17 (37.8)	2	0.006	25 (58.1)	18 (41.9)	4	0.015
	No	*n* = 125 (%)	100 (83.3)	20 (16.7)	5		91 (78.4)	25 (21.6)	9	
Coagulation	Yes	*n* = 78 (%)	54 (72.0)	21 (28.0)	3	0.136	50 (67.6)	24 (32.4)	4	0.210
	No	*n* = 94 (%)	74 (82.2)	16 (17.8)	4		66 (77.6)	19 (22.3)	9	
Respiratory	Yes	*n* = 14 (%)	10 (71.4)	4 (28.6)	0	0.519	8 (57.1)	6 (42.9)	0	0.702
	No	*n* = 158 (%)	118 (78.1	33 (21.9)	7		108 (74.5)	37 (25.5)	13	
Renal	Yes	*n* = 13 (%)	9 (69.2)	4 (30.8)	0	0.491	9 (69.2)	4 (30.8)	0	1
	No	*n* = 159 (%)	119 (78.3)	33 (21.7)	7		107 (73.3)	39 (26.7)	13	
Hepatobiliary	Yes	*n* = 6 (%)	3 (50)	3 (50)	0	0.127	3 (50)	3 (50)	0	0.345
	No	*n* = 166 (%)	125 (78.6)	34 (21.4)	7		113 (68.1)	40 (24.1)	13	

*Note*: Short‐term survival = survival until hospital discharge. Dogs that were euthanized due to financial restrictions or released against medical advice were excluded from this analysis. Long‐term survival = survival to ≥4 weeks after discharge. Dogs that were euthanized due to financial restrictions, released against medical advice, or had no follow‐up were excluded from this analysis. For the statistical analysis regarding the outcome, nonsurvival included both “euthanized” and “deceased” cases. Statistical significance was set at *p* ≤ 0.05.

Abbreviation: SIRS, systemic inflammatory response syndrome.

### 3.5 Blood Examinations

On Day 0, leukocytosis was present in 108 of 170 cases (63.5%), leukopenia in 47 of 170 cases (27.6%) (reference interval [RI]: 6.001–11.999 × 10^9^/L [6001–11,999/µL]), and a left shift in 53 of 96 cases (55.2%) (RI: 0–0.499 × 10^9^/L [0–499/µL]). Leukocytosis was diagnosed in 106 of 168 cases based on the blood count from Day 0. In two of the four cases, there was no blood count available for Day 0, but leukocytosis was present both on the previous and the next day, so it was assumed to have been present on Day 0 as well. Left shift was diagnosed in 52 of the 95 differential WBC counts by counting of bands. In one further case, a blood smear was analyzed, and a left shift was determined only subjectively. In 25 of the 76 cases with no information on Day 0, a differential WBC count was made on Day 1. Eight of these cases (32.0%) showed left shift. Low hematocrit was present in 99 of 168 cases (58.9%) (RI: 0.42–0.56 L/L [42%–56%]) and thrombocytopenia in 68 of 168 (40.5%) (RI: 165–400 × 10^9^/L [165,000–400,000/µL]). Canine C‐reactive protein (cCRP) was increased in 108 of 114 cases (94.7%) (RI: 0–10 mg/L [0–100 µg/mL]), and albumin concentration was increased in 4 of 136 (2.9%) and decreased in 80 (58.8%) (RI: 28–36 g/L [2.8–3.6 g/dL]). Lactate was increased in 58 of 104 cases (55.8%) (RI: 0–2.5 mmol/L [0–22.52 mg/dL]).

Regarding inflammatory markers, a difference was found between G1 and G2 in the leukocyte count on Day 0 (ANOVA, *p* < 0.001), with cases of nSIRS having higher leukocyte counts. No differences were detected between G2a, G2b, and G2c (ANOVA, *p* = 0.207) (Figure 1). No differences in cCRP concentrations were detected between G1 and G2 (ANOVA, *p* = 0.335) or G2a, G2b, and G2c (ANOVA, *p* = 0.969). Cases of G2 had lower serum albumin concentrations than G1 (ANOVA, *p* = 0.039), and no differences were detected between G2a, G2b, and G2c (ANOVA, *p* = 0.271) (Figure 2). Significantly higher lactate concentrations were found in cases of G1 compared with G2 (ANOVA, *p* = 0.033), but no differences were found between G2a, G2b, and G2c (ANOVA, *p* = 0.905).

**FIGURE 1:**
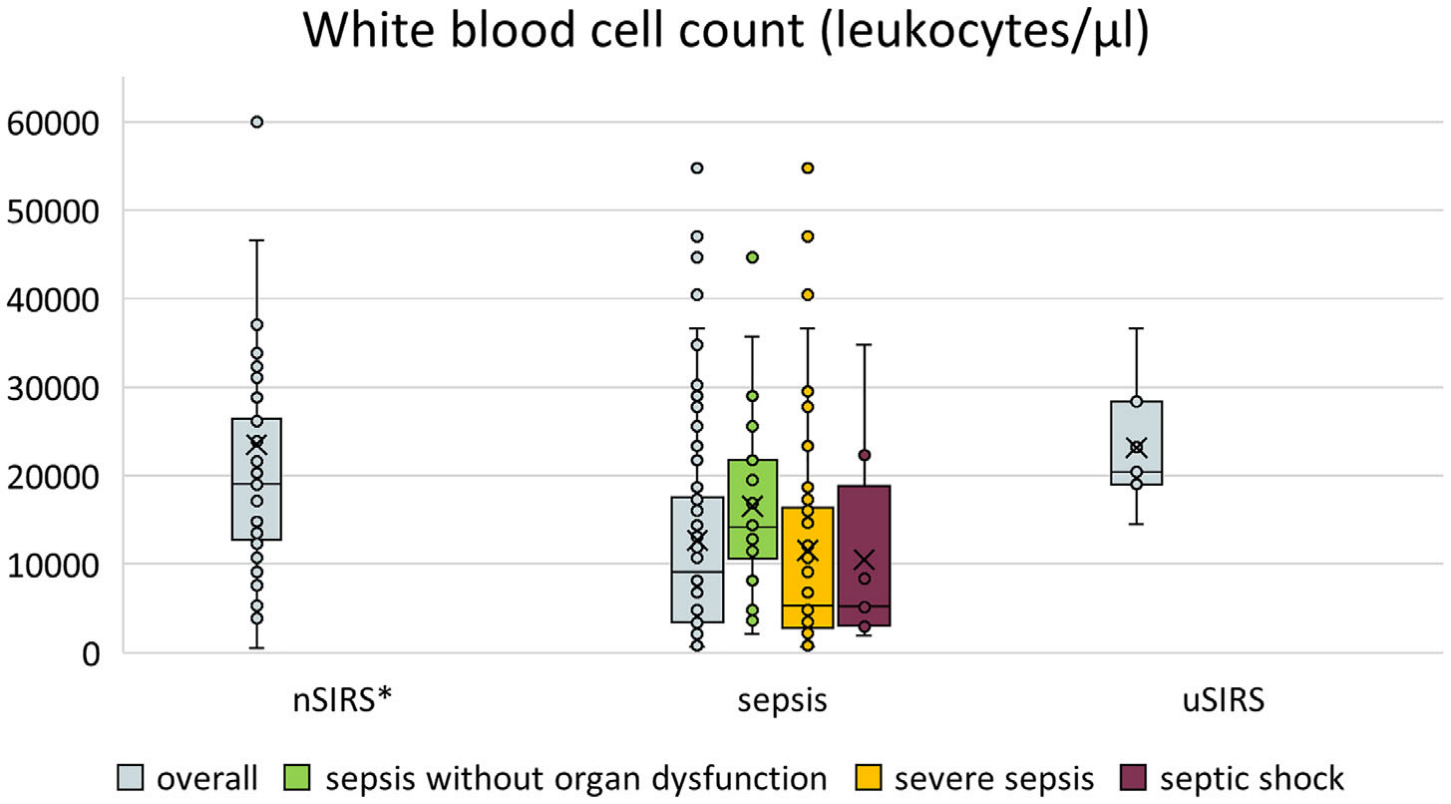
White blood cell count on Day 0 in 168 cases of SIRS or sepsis in dogs split into groups based on the origin and severity of SIRS or sepsis. Groups (G1, G2): *p* < 0.001. Subgroups (G2a–G2c): *p* = 0.207. *Three values are above the scale and are not shown here for reasons of presentation. G1, nonseptic SIRS; G2, sepsis; G2a, sepsis without organ dysfunction; G2b, severe sepsis (sepsis with organ dysfunction); G2c, septic shock; G3, SIRS of unknown origin; SIRS, systemic inflammatory response syndrome. Statistical significance was set at *p* ≤ 0.05.

**FIGURE 2:**
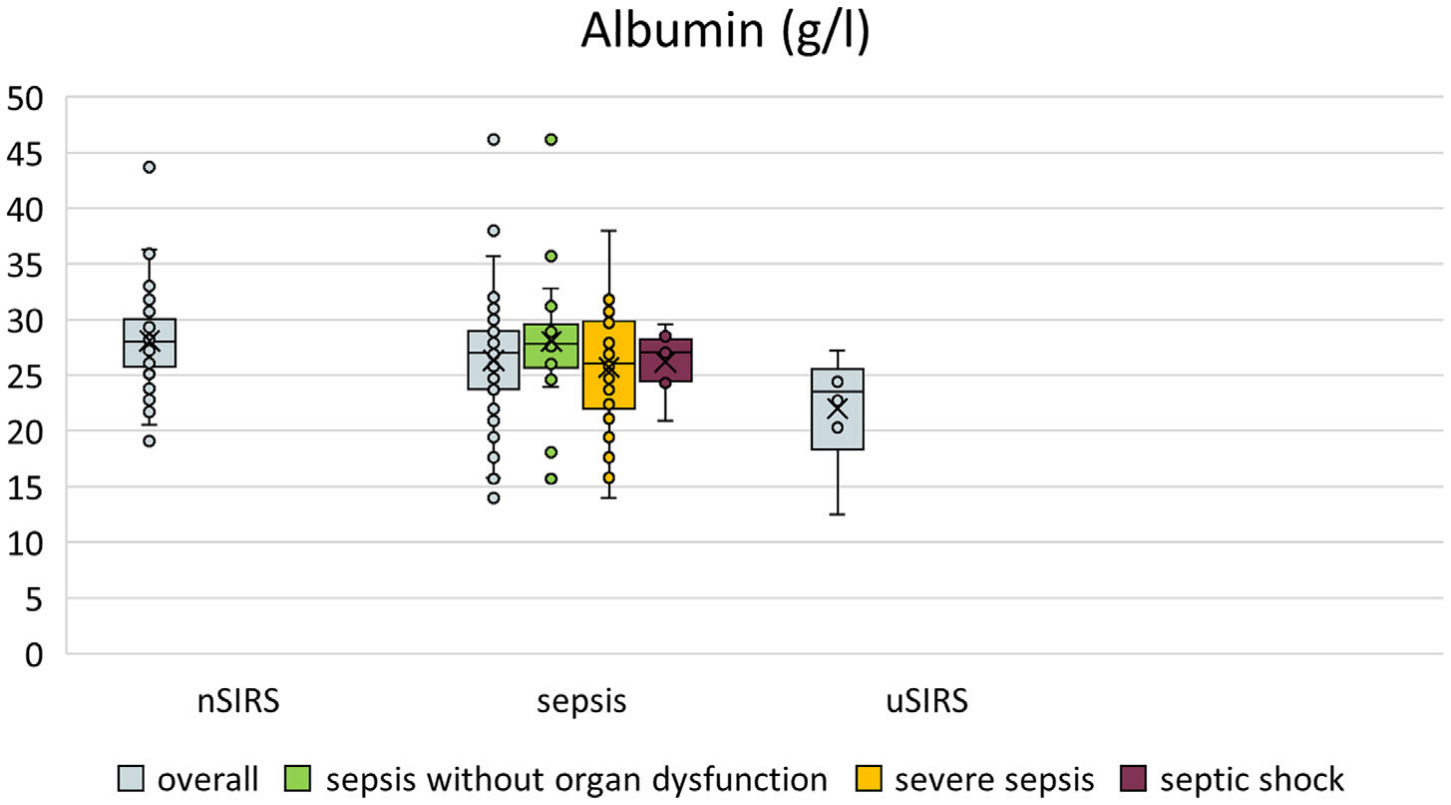
Serum albumin concentration on Day 0 in 138 cases of SIRS or sepsis in dogs split into groups based on the origin and severity of SIRS or sepsis. Groups (G1, G2): *p* = 0.039. Subgroups (G2a–G2c): *p* = 0.271. G1, nonseptic SIRS; G2, sepsis; G2a, sepsis without organ dysfunction; G2b, severe sepsis (sepsis with organ dysfunction); G2c, septic shock; G3, SIRS of unknown origin; SIRS, systemic inflammatory response syndrome. Statistical significance was set at p [less than or equal to sign] 0.05.

### 3.6 Microbiological Testing, Serology, and Cytology

Bacterial growth of clinically relevant pathogens could be detected in 22 cultures of different sample materials from 18 of 92 cases with sepsis (19.6%) (Table 5). Viral pathogens were detected in seven cases. One dog suffered from bacterial pneumonia (*Bordetella bronchiseptica*, *Mycoplasma cynos*) with an additional viral component (canine respiratory coronavirus). Parvovirus was detected in six cases (by PCR or SNAP‐test). The most common protozoal findings were *Babesia canis* (*n* = 23) and *Leishmania infantum* (*n* = 4).

**TABLE 5:** Bacterial pathogens detected by 22 cultures in 18 of 92 cases of sepsis in dogs.

Pathogen	Origin of sample							
	Blood/catheter	Respiratory tract/thorax/sputum	Abdominal organs/abdomen	Urine	Wound/abscess/infected eye	Reproductive tract	Gastrointestinal tract/feces	Oral cavity/pharynx/larynx
*Clostridium perfringens*	1							
*Escherichia coli*	1		2	1	1			
*Staphylococcus* spp.				1	2			
*Streptococcus canis*	1			1	1			
*Pasteurella canis*					1			
Mixed		2		1		1	3	2

*Note*: Cultures with bacterial growth without clinical relevance (i.e., contaminants) are not shown.

### 3.7 Treatment

One hundred thirty‐three cases (77.3%) were hospitalized, eight of which were ultimately referred and hospitalized elsewhere to complete treatment. The median duration of the hospitalization of the 125 remaining cases was 4 days (range: 1–19).

Among inpatients and outpatients combined, fluid therapy was administered in 154 cases (89.5%); in outpatients, this included short‐duration infusions administered at the clinic or via ambulatory infusion packs at home. Analgesics were provided in 138 cases (80.2%). Overall, 132 cases (76.7%) were treated with antimicrobial agents and 61 (35.5%) had been pretreated with antimicrobials according to the history. Two additional dogs had been pretreated with medication by a prior veterinarian, but the specific agent, whether antimicrobial or not, could not be determined. In the clinic, 117 cases (68%) were treated with the following antimicrobials (treatment continued from pretreatment, or started at our hospital): penicillins (*n* = 100), fluoroquinolones (*n* = 30), tetracyclines (*n* = 20), cephalosporins (*n* = 3), and aminoglycosides (*n* = 1). This count includes 33 cases that received two different classes of antimicrobials (penicillins and fluoroquinolones [*n* = 25]; penicillins and tetracyclines [*n* = 7]; fluoroquinolones and tetracyclines [*n* = 1]) and two cases that received three different classes of antimicrobials (penicillins, fluoroquinolones, and aminoglycosides [*n* = 1]; penicillins, fluoroquinolones, and cephalosporins [*n* = 1]). Noradrenalin as a vasopressor was administered in 10 cases (5.8% [*n* = 2 with nSIRS, *n* = 8 with sepsis]).

### 3.8 Outcome

One hundred twenty‐eight of 165 evaluable cases (77.6%) survived to discharge, and 116 of 159 (73%) survived at least four more weeks (i.e., long term). For the statistical analysis of short‐term survival, two dogs that were discharged against medical advice were excluded, in addition to five dogs that were euthanized due to financial restrictions. For the statistical analysis of long‐term survival, a further six cases were excluded because no follow‐up was possible.

## 4 Factors Associated With Outcome

### 4.1 SIRS Criteria and Outcome

No association was found with logistic regression between the number of fulfilled SIRS criteria and outcome (for short‐term survival: 2 vs. 3 criteria fulfilled, OR: 1.01, 95% CI: 0.41–2.44; 2 vs. 4 criteria fulfilled, OR: 2.39, 95% CI: 0.75–7.63; 3 vs. 4 criteria fulfilled, OR: 2.38, 95% CI: 0.72–7.81,; *p* = 0.245; for long‐term survival: 2 vs. 3 criteria fulfilled, OR: 1.48, 95% CI: 0.65–3.37; 2 vs. 4 criteria fulfilled, OR: 2.27, 95% CI: 0.70–7.30; 3 vs. 4 criteria fulfilled, OR: 1.54, 95% CI: 0.48–4.93; *p* = 0.148). Logistic regression found an association between the severity of leukocytosis and outcome. In patients with leukocytosis, nonsurvivors had higher leukocyte counts than survivors, with statistical significance only for long‐term survival (short‐term: *p* = 0.649; long‐term: *p* = 0.049). In patients with leukopenia, degree of leukopenia was not associated with survival (short‐term: *p* = 0.111; long‐term: *p* = 0.129).

### 4.2 Group and Outcome

A difference in survival was found between G1 and G2, with the better survival rate in G2 (short‐term: OR: 2.36, 95% CI: 1.06–5.24, Pearson *χ* ^2^ test, *p* = 0.046; long‐term: OR: 2.61, 95% CI: 1.22–5.56, Pearson *χ* ^2^ test, *p* = 0.015). Differences in survival between G2a, G2b, and G2c were observed for long‐term survival, with the best survival rate in G2a and the worst in G2c (short‐term: G2a vs. G2b OR: 3.59, 95% CI: 0.42–30.49; G2a vs. G2c OR: 16.39, 95% CI: 1.35–200; G2b vs. G2c OR: 4.59, 95% CI: 0.86–24.39, Fisher exact test, *p* = 0.060; long‐term: G2a vs. G2b OR: 4.67, 95% CI: 0.56–38.87; G2a vs. G2c OR: 15.87, 95% CI: 1.29–200; G2b vs. G2c OR: 3.38, 95% CI: 0.65–17.54, Fisher exact test, *p* = 0.044).

Because patients with babesiosis accounted for a large proportion of surviving patients with sepsis (Table 3), they were excluded from the outcome calculation on a trial basis. Under this condition, the survival rate for G2 was 82.8% (53/64) (instead of 86.2%) for short‐term survival and 79.0% (49/62) (instead of 83.3%) for long‐term survival. Excluding patients with babesiosis, there was no survival difference between G1 and G2 (short‐term: OR: 8.82, 95% CI: 0.79–4.16, Pearson *χ* ^2^ test, *p* = 0.219; long‐term: OR: 1.97, 95% CI: 0.90–4.32, Pearson *χ* ^2^ test, *p* = 0.120). Excluding dogs with babesiosis, differences in survival among G2a, G2b, and G2c remained different for short‐term and long‐term survival (short‐term: G2a vs. G2b OR: 6.52, 95% CI: 0.76–56.17; G2a vs. G2c OR: 10.99, 95% CI: 0.80–142.86; G2b vs. G2c OR: 1.69, 95% CI: 0.26–10.99, Fisher exact test, *p* = 0.050; long‐term: G2a vs. G2b OR: 8.75, 95% CI: 1.03–74.18; G2a vs. G2c OR: 10.53, 95% CI: 0.76–142.86; G2b vs. G2c OR: 1.20, 95% CI: 0.19–7.63, Fisher exact test, *p* = 0.043).

### 4.3 Underlying Diseases and Outcome

There was a difference in short‐ and long‐term survival among the disease categories (short‐term: Fisher exact test, *p* < 0.001; long‐term: Fisher exact test, *p* < 0.001), with the best survival rates for anaphylaxis, catheter‐associated infection, and wound/abscess. The worst short‐term survival was found in cases with neoplasia.

### 4.4 Organ Dysfunction and Outcome

Organ dysfunction had a significant impact on short‐term survival. Short‐term mortality was higher in cases with one or more organ dysfunctions (short‐term: OR: 2.57, 95% CI: 1.05–6.29, Pearson *χ* ^2^ test, *p* = 0.047) and was marginally higher in the long term with one or more organ dysfunctions (long‐term: OR: 2.23, 95% CI: 0.97–5.08, Pearson *χ* ^2^ test, *p* = 0.059) (Table 4). Moreover, logistic regression analysis found an association between a higher number of dysfunctional organ(system)s and worse outcome in patients with at least one dysfunctional organ system (short‐term: *p* = 0.019; long‐term: *p* = 0.022) (Table 4).

### 4.5 Blood Tests and Outcome

Anemia was more common in nonsurvivors than in survivors (short‐term: OR: 2.34, 95% CI: 1.04–5.24, Pearson *χ* ^2^ test, *p* = 0.039; long‐term: OR: 2.20, 95% CI: 1.03–4.72, Pearson *χ* ^2^ test, *p* = 0.046). Additionally, an increased lactate concentration was more common in nonsurvivors than in survivors (short‐term: OR: 3.00, 95% CI: 1.08–8.33, Pearson *χ* ^2^ test, *p* = 0.037; long‐term: OR: 1.57, 95% CI: 0.65–3.83, Pearson *χ* ^2^ test, *p* = 0.377), with statistical significance only for short‐term survival.

### 4.6 Treatment and Outcome

Hospitalization had an impact on outcome in that more hospitalized cases survived than nonhospitalized cases (short‐term: OR: 9.58, 95% CI: 4.09–22.46, Pearson *χ* ^2^ test, *p* < 0.001; long‐term: OR: 12.19, 95% CI: 5.04–29.47, Pearson *χ* ^2^ test, *p* < 0.001). In the nonhospitalized group, some dogs were so critically ill that they were euthanized or died shortly after presentation, and in such cases, hospitalization was not possible. The 37 nonsurvivors (short‐term) included 17 hospitalized (45.9%) and 20 nonhospitalized (54.1%) cases. The 128 survivors (short‐term) consisted of 114 hospitalized (89.1%) and 14 nonhospitalized (10.9%) cases. Nineteen of the nonhospitalized nonsurvivors were euthanized on Day 0; one died naturally, and three of the hospitalized nonsurvivors died naturally or were euthanized on Day 0, despite intensive medical care. In logistic regression analysis, the length of inpatient stay of hospitalized patients did not differ between survivors and nonsurvivors (short‐term: *p* = 0.089; long‐term: *p* = 0.568).

## 5 Discussion

The most common causes of SIRS or sepsis in dogs in this study population were diseases of the gastrointestinal tract, followed by vector‐borne diseases and neoplasia. As these are frequently diagnosed, serious clinical conditions that often lead to the death of the affected individual [17, 18, 19, 20, 21, 22, 23, 24], identifying the causes and factors that influence the outcome in these patients is essential. Variable cutoff points within the four general SIRS criteria have been used to diagnose SIRS in dogs [6, 7, 30]. For better comparison of study results, standardized values should be established in veterinary medicine. Furthermore, some of the SIRS criteria can also be altered due to physiologic conditions, such as anxiety and physical activity [41, 42]. Therefore, it is important to restrict their use to critically ill patients with an underlying disease that could be the cause of the variations [1].

It has been proposed that SIRS could be graded as mild, moderate, and severe depending on how many criteria are met [6, 43]. Similar to other studies, an association between the number of fulfilled SIRS criteria and outcome was not found in our cohort [27, 44]. However, in this study population, cases with organ dysfunction (which were found to have a poorer outcome) fulfilled more SIRS criteria. Further studies are needed to determine whether there is an association between the number of SIRS criteria met and disease severity.

Group allocation was done as accurately as possible based on all available results. Due to the observational nature of the study, not all patients underwent the same diagnostic tests. Some patients died prematurely due to their poor clinical condition, or tests were not performed due to financial restrictions, resulting in less information available in some cases. In addition, it cannot be ruled out that in some cases, infectious agents were not detected (e.g., due to antimicrobial pretreatment) [22, 45, 46, 47, 48].

In the literature, there are different findings regarding whether dogs with sepsis or with nSIRS have better chances of survival [18, 19, 21]. It is conceivable that the underlying disease and its treatability have a bigger influence on the outcome than the presence of an infectious agent itself. In our study cohort, cases suffering from sepsis were found to have better survival than cases with nSIRS. Vector‐borne diseases were the most frequent cause of sepsis, in which a good survival rate was observed. Babesiosis was the most commonly diagnosed vector‐borne disease, for which recent studies have previously shown a high survival rate if treated properly [49, 50]. The difference in survival between G1 and G2 was no longer statistically significant when cases of babesiosis were excluded, exemplifying their influence on the results.

Organ dysfunction was found in 66.3% of the total study population and in 72.8% of cases with sepsis (Table 4). A similar or slightly higher occurrence of organ dysfunction (66%–90.6%) has been found in recent studies [22, 23, 24, 38]. Variations can be explained by different disease severity in the patient populations and by differences in the definitions of organ dysfunction, the presence of which was a negative prognostic factor for survival in this study population. Similar results were published in a study by Kenney et al. [24] in which a negative association was found between the number of affected organ system(s) and survival. Various findings have been published in recent studies, with some identifying an association [22, 23, 24], while one study did not demonstrate such a relationship [27].

cCRP, as a major acute‐phase protein in dogs, is an important marker for inflammation [21, 51, 52, 53]. Because cCRP rises more quickly than the leukocyte count and can be increased in cases with normal leukocyte count or leukopenia, it can be helpful for the rapid identification of inflammatory disease [53]. As described previously [21], we also found that differentiation between nSIRS and sepsis based on cCRP was not possible, and that the cCRP concentration measured on Day 0 is not itself an adequate prognostic parameter.

In the current study, we found that hospitalization had a positive impact on outcome. This finding has risk of bias, because many nonsurvivors were euthanized at presentation due to critical status and, therefore, were not hospitalized. The categorization of reasons for euthanasia was performed as accurately as possible. Important aspects for decision‐making were the dog's clinical condition, financial constraints, and the dog's prognosis if treated with adequate therapy. The short‐term survival rate of 77.6% corresponded to other veterinary studies that reported survival rates between 59.3% and 78.9% [17, 19, 20, 51, 54]. The long‐term survival rate of 73% was higher than documented rates reported in other studies (34.0%–49.3%) [51, 55]. Variations in survival rates may be explained by the fact that there is no consensus regarding the value intervals that should be applied to the SIRS criteria in dogs; therefore, included cases may differ in illness severity among veterinary studies. It is not possible to eliminate all bias regarding treatment and outcome within the study population due to differences in client resources and decision‐making.

The SIRS criteria are criticized for their lack of sensitivity and specificity [4, 7, 17, 48, 56, 57]. Fulfillment of criteria can indicate inflammation but may not necessarily reflect a dysregulated, life‐threatening host response to inflammation [4, 58]. Nevertheless, SIRS criteria have been used commonly to diagnose veterinary patients with SIRS and sepsis [1, 22, 27, 59, 60]. Using the new definitions with the Sequential Organ Failure Assessment score to diagnose sepsis is difficult to implement in veterinary medicine, as some of the required parameters (e.g., urine output, MAP) are not routinely evaluated. Unfortunately, the quick Sequential Organ Failure Assessment score yielded less reliable results in canine populations [61, 62]. Furthermore, the use of different criteria would have reduced comparability with previous studies. Therefore, a limitation of this study, as with most other veterinary studies, remains the use of SIRS criteria designed for people for the diagnosis of SIRS and sepsis.

Because this study was conducted at a university hospital, many patients were included that were referred by private practitioners. The frequency of the underlying diseases may thus be influenced by the expertise of our hospital's veterinarians as well as that of the referring veterinarians. Furthermore, geographical bias should be noted. For example, babesiosis was diagnosed frequently in Berlin/Brandenburg in recent years [49].

This observational study confirms that SIRS can be triggered by a variety of diseases in dogs. Cases with organ dysfunction were found to have a lower probability of survival. Because of this, close, routine monitoring is important to identify organ dysfunctions at an early stage in an attempt to improve patient outcome.

## Author Contributions

**Myriam A. Knopf**: conceptualization, data curation, formal analysis, investigation, methodology, project administration, validation, visualization, writing – original draft. **Christiane Weingart**: conceptualization, data curation, formal analysis, methodology, investigation, supervision, validation, visualization, writing – review and editing. **Roswitha Merle**: conceptualization, formal analysis, methodology, visualization, writing – review and editing. **Teresa Sandbrink**: conceptualization, data curation, investigation, methodology, validation, visualization, writing – review and editing. **Antina Lübke‐Becker**: conceptualization, data curation, methodology, writing – review and editing. **Marcus Fulde**: conceptualization, methodology, writing – review and editing. **Caroline Robé**: conceptualization, methodology, visualization, writing – review and editing. **Marianne Skrodzki**: conceptualization, investigation, methodology, writing – review and editing. **Uwe Rösler**: conceptualization, methodology, writing – review and editing. **Stefan Schwarz**: conceptualization, methodology, visualization, writing – review and editing. **Barbara Kohn**: conceptualization, data curation, funding acquisition, formal analysis, investigation, methodology, project administration, resources, supervision, validation, visualization, writing – review and editing.

## Funding

Myriam A. Knopf declares that she was supported by a scholarship from the Ernst Reuter Foundation. We acknowledge support by the Open Access Publication Fund of the Freie Universität Berlin.

## Disclosure

Partial results have been presented at the 69th annual congress of the German Society for Small Animal Medicine (DGK‐DVG), the 32nd Annual Conference of the DVG Division of Internal Medicine and Clinical Laboratory Diagnostics (InnLab), and the 34th ECVIM‐CA annular congress.

## Ethics Statement

The authors confirm that the journal's ethical guidelines have been followed. The authors state that no ethical approval was required for this study in accordance with local legislation and institutional requirements due to its observational character. No written informed consent was obtained from the animal owners because it was not required. No patients or clients are identifiable.

## Conflicts of Interest

Myriam A. Knopf, Marcus Fulde, Antina Lübke‐Becker, Roswitha Merle, Caroline Robé, Uwe Rösler, Teresa Sandbrink, Stefan Schwarz, and Marianne Skrodzki declare no conflicts of interest. Christiane Weingart declares that she has given lectures for veterinary pharmaceutical and diagnostics companies. Barbara Kohn has held lectures, had research collaborations, and acted as a consultant for veterinary pharmaceutical and diagnostic companies.

## Endnotes

1 Model 811‐B, Parks Medical Electronics, Aloha, OR.

2 Vet20, SunTech Medical, Morrisville, NC.

3 day service: SYSMEX XT‐2000i, Sysmex Deutschland GmbH, Norderstedt, Germany, January 1, 2023, to September 14, 2023.

4 day service: XN‐VET, Sysmex Deutschland GmbH, Norderstedt, Germany, September 15, 2023, to December 31, 2023.

5 emergency service: VetScan, scil animal care company GmbH, Viernheim, Germany, January 1, 2023, to October 31, 2023.

6 emergency service: Element HT5, scil animal care company GmbH, Viernheim, Germany, November 1, 2023, to December 31, 2023.

7 May‐Grünwald‐Giemsa staining according to Pappenheim, Sigma‐Aldrich Chemie GmbH, Taufkirchen, Germany.

8 Diff‐Quick staining, Labor + Technik, Berlin, Germany.

9 day service: Konelab Prime 60i, Thermo Fisher Scientific Inc., Vantaa, Finland, January 1, 2023, to October 29, 2023.

10 day service: Indiko, Thermo Fisher Scientific Inc., Vantaa, Finland, October 30, 2023, to December 31, 2023.

11 emergency service: GEM Premier 3500, Werfen GmBH, Munich, Germany, January 1, 2023, to December 31, 2023.

12 emergency service: Catalyst‐One, IDEXX Laboratories, Inc., Westbrook, ME, January 1, 2023, to December 31, 2023.

13 emergency service: scil Reflovet Plus, scil animal care company GmbH, Viernheim, Germany, January 1, 2023, to December 31, 2023.

14 DIAGNOSTICA STAGO S.A.S., Asnières‐sur‐Seine, France.

15 Siemens Healthcare Diagnostic Products GmbH, Marburg, Germany.

16 according to Schnitger and Gros, Amelung, Lemgo, Germany.

17 DigitalDiagnost, Philips, Hamburg, Germany

18 LOGIQ S7, Scil animal care company GmbH, Viernheim, Germany.

19 Vivid S60N, GE Vingmed Ultrasound AS, Horten, Norway.

20 PC EKG 2000, Eickemeyer, Tuttlingen, Germany.

21 CP 50, Welch Allyn, Navan, Ireland.

22 Combur^9^Test strips, Roche Holding, Basel, Switzerland.

23 A.KRÜSS Optronic GmbH, Hamburg, Germany.

24 Laboklin, Bad Kissingen, Germany.

25 IVD GmbH, Seelze‐Letter, Germany.

26 Institute for Experimental Parasitology, LMU Munich, Germany.

27 IDEXX Laboratories, Kornwestheim, Germany.

28 IBM SPSS Statistics 29.0.0.0/29.0.2.0 for Windows, Chicago, IL.

29 Microsoft Word for Microsoft 365, Microsoft Corporation, Redmond, WA.
